# Effects of supplementation of probiotics instead of antibiotics to broiler diet on growth performance, nutrient retention, and cecal microbiology

**DOI:** 10.5455/javar.2021.h543

**Published:** 2021-09-29

**Authors:** Md. Mustafijur Rahman, Mohammad Mehedi Hasan Khan, Md. Matiar Rahman Howlader

**Affiliations:** 1 Department of Physiology, Sylhet Agricultural University, Sylhet, Bangladesh; 2 Department of Biochemistry and Chemistry, Sylhet Agricultural University, Sylhet, Bangladesh

**Keywords:** Probiotics, *Bacillus subtilis*, *Saccharomyces boulardii*, growth performance, nutrient retention, cecal microbiology

## Abstract

**Objectives:**

The research was carried out on broilers to determine the efficacy of probiotics (*Bacillus subtilis* and *Saccharomyces boulardii* combined) supplementation on growth performances, nutrient retention (metabolizable energy, dry matter, and crude protein), and cecal microbiology (*Bifidobacteria *spp., *Clostridium *spp., and coliforms).

**Materials and Methods:**

A total of 160 broiler chicks (day-old) were selected and differentiated randomly into 4 groups (T_0_, T_1_, T_2_, and T_3_) (40 × 4) comprising 40 birds in every single group. The control group (T_0_) was fed commercial broiler feed only and the other three groups, referred to as treatment groups (T_1_, T_2_, and T_3_), were treated with 1 gm ciprofloxacin, 1 gm probiotic, and 1 gm probiotic plus 0.5 gm enzyme, respectively, in per liter of fresh dietary water 8 h daily for 7 days in each phase. Experimental trials were divided into 2 phases, the starter phase from day 0 to 21 and the finisher phase from day 22 to 35.

**Results:**

Bodyweight gain and nutrient retention in experimental broiler birds in treatment groups were significantly (*p *< 0.05) higher than the control group. Overall body weight gain and nutrient retention of broiler chicks in treatment groups T_2 _and T_3_ were better than T_1_. From day 22 to 35, cecal *Clostridium *and coliform bacterial load counts were significantly lower *p *< 0.01, *p *< 0.05, and *p *< 0.01, respectively, in T_1_, T_2_, and T_3 _treatments than T_0_. Overall, *Clostridium *and coliform bacterial counts in the birds of treatment group T_2_ were significantly lower (*p *< 0.05) than T_0_.

**Conclusion:**

The probiotics, in addition to enzyme supplementation, had suitable influence effects on growth performance of broilers, birds retention of nutrient, and microfloral count in birds’ cecum.

## Introduction

Livestock added 1.54% of total gross domestic product (GDP) and 3.40% in GDP growth in Bangladesh for the fiscal year 2017–18 [1]. The protein intake by a human is 55.04 gm/day, in which animal protein provides 9.6 gm against the Food and Agriculture Organization (FAO) recommendation of 28 gm [2] in Bangladesh. Chickens are more susceptible to growth retardation, malnutrition, and digestive problem due to harmful gut flora, reduced absorption, and retention of nutrients affecting the optimum production. Many farmers use vast amounts of antibiotics haphazardly, which has health hazards to the consumer and broiler industry. The growing picture of antibiotic resistance in broiler birds and humans due to its excessive and uncontrolled use and improper maintenance of the withdrawal period of antibiotics in the poultry sector has recently been a significant public health issue. Due to this aspect, antibiotics in livestock and poultry have been strongly limited or banned in many nations, including the European Union, since 2006. Still, in Bangladesh, it is yet to be established. Considering this present situation, an emergency need is felt to find an alternative of antibiotics for better health and production of poultry in commercial raring [3]. Probiotics are suitable for filling this gap at the farmers level in preference to antibiotics [4,5]. Probiotics are readily available and widely used at the field level to improve growth performance [6], nutrient retention [7–9], cecal microbial balance [10,11], and intestinal morphology [6]. As probiotics, *Bacillus *spp. is preferred because of its higher resistance spores and long durability in the natural environment [12,13]. Different studies have stated that the solid substrate fermentation method of probiotic production is cost-effective and suitable for the environment [14].

Enzymes may favor the growth of probiotic organisms and improved performance during fermentation [15] by *Bacillus subtilis* and *Saccharomyces boulardii*. It might reduce the production cost of probiotics and decrease environmental pollution by culturing in the laboratory. Therefore, the present research work has been carried out to measure the efficacy of using probiotics on broiler growth performance, nutrient retention, and cecal microbiology.

## Materials and Method

### Ethical approval

The research was carried out at the Department of Physiology and Central Laboratory, Sylhet Agricultural University, Sylhet-3100, Bangladesh. The use and care of poultry and animals followed the guidelines of the National Research Council (NRC) for research. The ethical approval committee for research animal care and use of Sylhet Agricultural University, Sylhet-3100, Bangladesh, gave permission for this work (Permit #AUP2017001).

### Experimental birds, diets, and management during the study period

A total of 160, day-old broiler chicks were kept well-ventilated, hygienic, and in proper atmospheric conditions. The experimental birds were fed broiler starter and finisher ration during the whole study period. Random selection and differentiation into 4 groups of broiler birds consisted of 40 birds in each group, depending on the initial body weight in a randomized block design (RBD). Every treatment group had 40 broiler birds assigned to 4 replications of 10 in each. Dietary treatments were as follows:

Group T_0_: Supplied commercial feed of broiler and drinking water.

Group T_1_: 1 gm antibiotic (ciprofloxacin) per liter of fresh drinking water without any treatment + broiler ration 8 h daily for 7 days in each phase.

Group T_2_: 1 gm probiotics (Promax) per liter of fresh drinking water without any treatment + broiler ration 8 h daily for 7 days each phase.

Group T_3_: 1 gm probiotic addition with 0.5 gm commercial enzyme (polyzyme) per liter fresh drinking water 8 h daily for 7 days in each phase.

In this study, 1 gm *B. subtilis *and *S. boulardii* were supplemented because of the best efficacy of probiotics. Commercial broiler pellet feed is recommended for the starter phase and finisher phase. The used antibiotic (ciprofloxacin) was added to the feed to compare with probiotics, and probiotics were supplemented to the two-phase-starter phase and finisher of the experimental trial. All the essential nutrients were supplied according to the nutrient requirements recommended by the NRC in 1994. Rice husk was used as a bedding material on the floor of the birds’ houses. Separate self-feeder and cup drinker were used in each cage to provide easy access to water and feed. For the first 5 days, the temperature at the broiler shed was regulated at 34°C and then decreased gradually as standard management. Lighting was provided to the broiler in each group for 14 h/day.

### Sampling and measurement

The live weight of experimental broiler chickens was weighed at the two phases with 15-day intervals with the help of weighing balance. Two birds from each cage were placed in an individual cage (single bird/cage) from day 14 (starter phase) and 28 (finisher phase) onwards for the collection of cecal samples. The cecal samples (50 gm/bird/day) were collected for the last 48 h in every single phase and placed into a plastic jar for further processing. Eight birds from each treatment group (two from each cage) were randomly selected and sacrificed at the end of the starter phase on day 21 and the finisher phase on day 35. The cecum of slaughtered birds was opened up to collect the cecal samples. The collected cecal contents were placed in separately marked sterile plastic bottles for each bird with phosphate buffer solution preserved on an ice bucket until the analysis.

### Chemical composition and microbial population analysis

Dry Matter (DM) and Crude Protein (CP) content of birds’ feed and cecal samples were analyzed by the AOAC International method [16]. Metabolizable Energy (ME) values were calculated following the procedure of Sakomura and Rostagno [17]. The retention of DM, CP, and ME percentage were calculated by subtracting the DM, CP, and ME percentage in feces from the DM, CP, and ME percentage intake by birds through the feed. The population of the cecum was analyzed by following the procedure of Choi et al. [18]. The analyzed microbial groups were differentiated into the following parameters: total anaerobic bacterial count was evaluated using Tryptic Soy Agar (Man, Rogosa and Sharpe (MRS) agar + 0.02% NaN3 + 0.05% L-cystine hydrochloride monohydrate) used in *Bifidobacterium *spp. count, and Violet Red Bile agar used for the counting coliforms. *Clostridium *spp. count was carried out in Tryptose Sulphite Cycloserine agar.

### Statistical analysis

The expected data observed were input into an MS Excel Worksheet, arranged and prepared for statistical analysis. One-way ANOVA was carried out through the statistical software (1996).

## Results

### Effect of treatments on growth performance

The live weight gain of experimental birds did not differ significantly from treatments on day 1 (beginning of the experiment). During the starter, finisher, and almost throughout the study period, the birds of T1, T2, and T_3_ groups showed significantly (*p *< 0.05; Table 1) better FCR than birds of group T_0_. During the study period, the birds of T_1_ group showed a higher gain of body weight and better feed conversion ratio (FCR) than the birds in group T_0_ but lower than the birds in T_2_ and T_3_ groups. The FCR of group T_3 _birds was significantly higher (*p *< 0.05) compared to other groups in the finisher stage. It showed better FCR and growth performances during the study period, and FCR increased significantly (*p* < 0.05) in the finisher stage due to probiotics supplementation.

### Effect on nutrient retention

The retention of nutrients such as DM and ME of T_1_ group on day 21 of the experiment was almost similar to group T_0_, and the retention of CP and ME was improved (Table 2) in the birds of T_1_, T_2,_ and T_3 _groups, respectively, than the birds in T_0_ group, but did not differ significantly. The retention of DM, CP, and ME at the finisher stage (day 22–35) in treated birds were better than in the control group. The DM retention differed significantly (Table 2; *p* < 0.01) in the treated birds’ group compared to the control. However, the retention of DM, CP, and ME of experimental birds in group T_3_ was relatively higher than that of other groups during the study period.

### Effect on cecal microbiology

In the cecum, *Bifidobacteria *spp. among the experimental treatments at day 21 in the birds of T_1_, T_2_, and T_3_ groups recorded higher than the birds in T_0_ group (Table 3). On the other hand, the cecal *Clostridium* and coliforms counts were decreased in the T1, T2, and T3 birds than the birds of T_0_ group (*p *< 0.05, Table 3). However, on day 35, the birds that were supplemented with antibiotic, probiotic, and enzyme (T_1_, T_2_, and T_3_, respectively) diets showed significantly lower loads (*p *< 0.01) in cecal *Clostridium *and coliform compared to the birds in T_0_. On day 35, the beneficial *Bifidobacterium* showed higher counts in the birds of T_2_ and T_3_ than the birds in other groups. *Clostridium *and coliform bacterial load counts of T_2_ and T_3_ showed highly significantly lower (*p *< 0.01) than birds of T_0_ and T_1 _(Table 3). Overall, the birds in T_3_ showed higher beneficial bacterial count and decreased harmful bacteria count during the study period.

## Discussion

The efficacy depends on various factors such as bacterial strain, dose, method of administration, survival capacity to a harsh environment, viability in storage for longer period, fermentation, and substrate used [14]. The most widely used probiotic microbe is *B. subtilis*, which is resistant to harsh storage conditions and higher temperatures. These bacteria are spore-formers and are generally considered safe strain as probiotics. Several favorable results have been found to use probiotics supplementation with feed or water for different poultry species using various strains of *Bacillus *[19]. The primary motto of this research was to determine the efficacy of probiotics on growth performances, nutrient retention, and cecal microbiology in broiler chicken. Ciprofloxacin was used to evaluate the potentiality of *B. subtilis *and *S. boulardii* as probiotics and alternatives to antibiotics. The enzyme was used to promote the growth of the microbes through digestion, absorption, and growth performances of the broiler.

**Table 1.** Effects of antibiotics and probiotics treatment on broiler growth performance.

Parameter	T_0_	T_1_	T_2_	T_3_	SEM	*p*-value
Starter (day 0–21)						
Weight gain (kg)	1.0800	1.0900	1.2100	1.3300	0.0459	0.148
Feed intake (kg)	1.7500	1.6900	1.9100	2.1500	0.0839	0.208
FCR	1.6200	1.5505	1.5785	1.6135	0.0148	0.369
Finisher (day 22–35)						
Weight gain (kg)	1.1000	1.1250	1.1500	1.2000	0.0328	0.815
Feed intake (kg)	2.0900	2.0800	2.0700	2.1000	0.0560	0.999
FCR	1.9000^a^	1.8480^ab^	1.8005^ab^	1.7480^b^	0.0226	0.024*
Overall (day 0–35)						
Weight gain (kg)	2.1800	2.2150	2.3600	2.5300	0.0728	0.363
Feed intake (kg)	3.8400	3.7790	3.9800	4.2500	0.1230	0.639
FCR	1.7610	1.7015	1.6860	1.6775	0.0146	0.139

SEM = Standard error of means.

abc Values with different superscripts in the same row differ significantly.

** 1% level of significance (*p *<0.01).

* 5% level of significance (*p *< 0.05).

**Table 2.** Effects of antibiotics and probiotics treatment on broiler nutrient retention.

Parameter	T_0_	T_1_	T_2_	T_3_	SEM	*p*-value
Starter (day 0–21)						
DM%	75.100	75.400	76.700	77.100	0.467	0.427
CP%	62.900	63.600	65.500	65.850	0.599	0.238
ME%	76.400	76.500	77.000	77.650	0.425	0.811
Finisher (day 22–35)						
DM%	76.300^a^	77.950^ab^	78.600^b^	79.700^c^	0.475	0.003**
CP%	64.00	64.700	65.200	65.700	0.305	0.248
ME%	78.800	79.600	79.800	80.100	0.222	0.177

SEM = Standard error of means.

abc Values with different superscripts in the same row differ significantly.

** 1% level of significance (*p *<0.01).

* 5% level of significance (*p *< 0.05).

**Table 3.** Effects of antibiotics and probiotics treatment on broiler cecal microbiology (CFU/gm).

Parameter	T_0_	T_1_	T_2_	T_3_	SEM	*p*-value
Starter (day 0–21)						
*Bifidobacteria *spp. (1 × 10^4^)	2.250	2.450	2.500	2.550	0.073	0.595
*Clostridium *spp. (1 × 10^4^)	2.550^a^	2.100^b^	1.850^b^	2.000^b^	0.101	0.005**
*Coliforms *spp. (1 × 10^4^)	1.800	1.700	1.700	1.450	0.073	0.452
Finisher (day 22–35)						
*Bifidobacteria spp. *(1 × 10^4^)	2.100^a^	2.500^a^	2.750^ab^	2.950^b^	0.124	0.006**
*Clostridium *spp. (1 × 10^4^)	2.250^a^	2.150^ab^	1.950^ab^	1.550^b^	0.110	0.039*
*Coliforms *(1 × 10^4^)	1.850^a^	1.550^b^	1.500^b^	1.350^b^	0.070	0.005**

SEM = Standard error of means.

abc Values with different superscripts in the same row differ significantly.

** 1% level of significance (*p *<0.01).

* 5% level of significance (*p *< 0.05).

This study found that supplemented probiotic to broiler diet improved growth at the starter stage to 21 days. The findings agreed with the report of Bai et al. [20], who explained that body weight gain of broiler birds increased by feeding probiotics at 0.1%–0.3% dose level during the starter phase (1–21 days) [20]. It is, therefore, recommended that supplementing 1 gm probiotic product used in each kilogram diet instead of antibiotic for highest production performance of broilers chicks which is similar to the findings of previous researches [10,21], demonstrating that average body growth and FCR was better at 1–21 days with the supplementation of 0.1% *Lactobacillus *spp., but not in the finisher phase (22–42 days). Supplementation of *B. subtilis *with enzyme resulted in improved body growth, FCR, and intake of feed. The efficacy of using probiotic (*B. subtilis*) to broiler diet was found similar to a previous report [22]. During the whole study period, the birds treated with *B. subtilis *and *S. boulardii *and enzyme showed comparatively better FCR than those supplementing with antibiotic ciprofloxacin. The live weight and FCR were higher in birds treated with *B. subtilis* and *S. boulardii* along with the enzyme than in other groups [23,24]. The growth performance of experimental birds was recorded higher in probiotic treatment than the birds that received the antibiotic. It might be due to an increased amount of nutrient retention and an improved gut microbial environment. 

This study found higher nutrient retention such as DM, ME, and CP among different treatment groups of birds supplemented with probiotics (*B. subtilis* and *S. boulardii*) than birds supplemented with antibiotics. The findings of this study are in agreement with Shim et al. [14], who revealed that the nutrient retention was highest among the probiotics-treated birds than control antibiotics-treated group. Retention of nutrients was higher in the birds treated with probiotics. It might be due to improved beneficial intestinal microbes and barrier function that decreased pathogenic microorganisms, increased functional intestinal microbial balance, and stimulated mucosal immune system [25]. Previously, higher nutrient retention and growth performances were reported in antibiotic supplementation [26]; however, it was not better than probiotics. The primary reason for the increased body weight gain observed in broiler chicks fed with probiotics during the starter phase is believed to be increased feed nutrient retention and digestibility.

Birds treated with probiotics (*B. subtilis* and *S. boulardii*) showed a significant decrease in harmful micro-flora like *Clostridium *and coliform*s *in cecal content at day 35. Previously, many research reports showed that supplementation of various probiotics in broiler chicken can decrease the pathogenic bacterial population in the gut and replace intestinal microflora with beneficial bacteria [25]. Probiotics assist treated animals by promoting a healthy intestinal environment [3] and microbial population balance [14,26]. They do this by increasing beneficial microorganisms and lowering harmful microbes. The findings of this study indicate that supplementing broiler chicks’ drinking water with probiotics improved body growth, nutrient retention, and cecal microbiota.

## Conclusion

From this research work, probiotic supplementation to broiler ration and its influence on growth performance, cecal microbiology, and retention of nutrients were determined and compared instead of using antibiotics on broiler. This research work also showed that supplementation of probiotics to broiler diet is essential to improve body weight, feed intake, better FCR, increase retention of nutrients, and improve the gut flora condition in broilers.

## List of abbreviations

FCR, Feed conversion ratio; MRS, Man, Rogosa and Sharpe; FAO, Food and Agriculture Organization; DM, Dry matter; CP, Crude protein; ME, Metabolizable Energy; NRC, National Research Council; RBD; Randomized block design.
